# Rapid Characterization of Black Tea Taste Quality Using Miniature NIR Spectroscopy and Electronic Tongue Sensors

**DOI:** 10.3390/bios13010092

**Published:** 2023-01-05

**Authors:** Guangxin Ren, Xusheng Zhang, Rui Wu, Lingling Yin, Wenyan Hu, Zhengzhu Zhang

**Affiliations:** 1 School of Biological Engineering, Institute of Digital Ecology and Health, Huainan Normal University, Huainan 232038, China; 2 State Key Laboratory of Tea Plant Biology and Utilization, Anhui Agricultural University, Hefei 230036, China; 3 Key Laboratory of Bioresource and Environmental Biotechnology of Anhui Higher Education Institutes, Huainan Normal University, Huainan 232038, China; 4 Library, Huainan Normal University, Huainan 232038, China

**Keywords:** tea, near-infrared spectroscopy, electronic tongue, chemometrics, quality control

## Abstract

The taste of tea is one of the key indicators in the evaluation of its quality and is a key factor in its grading and market pricing. To objectively and digitally evaluate the taste quality of tea leaves, miniature near-infrared (NIR) spectroscopy and electronic tongue (ET) sensors are considered effective sensor signals for the characterization of the taste quality of tea leaves. This study used micro-NIR spectroscopy and ET sensors in combination with data fusion strategies and chemometric tools for the taste quality assessment and prediction of multiple grades of black tea. Using NIR features and ET sensor signals as fused information, the data optimization based on grey wolf optimization, ant colony optimization (ACO), particle swarm optimization, and non-dominated sorting genetic algorithm II were employed as modeling features, combined with support vector machine (SVM), extreme learning machine and *K*-nearest neighbor algorithm to build the classification models. The results obtained showed that the ACO−SVM model had the highest classification accuracy with a discriminant rate of 93.56%. The overall results reveal that it is feasible to qualitatively distinguish black tea grades and categories by NIR spectroscopy and ET techniques.

## 1. Introduction

Black tea is the most widely distributed and consumed tea product worldwide and is one of the most important fermented beverages in the world [1]. According to the different processing techniques and shape characteristics, black tea can be divided into Congou black tea, Souchong black tea, and Broken black tea. Among them, Congou black tea is a famous tea product in China, its shape is intact, and the branches are tight and thin, which are processed through four processes, namely withering and losing water in fresh leaves, kneading and forming, fermentation and drying [2]. Compared to Congou black tea, broken black tea is mainly processed with the fresh leaves of large-leaf species, and the kneading process is replaced by kneading and cutting, resulting in a more fragmented or granular shape, which is more conducive to the rapid leaching of nutrients. The main flavor-presenting substances in black tea are tea polyphenols, catechins, free amino acids, caffeine, soluble sugars, inorganic salts, and mineral elements, which are considered to be the main sources of astringency, bitterness, sweetness, and saltiness in black tea, and the composition and proportions of the above active ingredients determine the diversity of the tea’s taste [3,4]. To guide consumers in making the right purchasing decisions, prevent fraud, and avoid financial losses, a quick and effective evaluation of the quality of different grades and flavors of black tea has become a pressing issue.

At present, the traditional methods for analyzing and evaluating the quality of tea and its products (i.e., sensory quality evaluation and physical and chemical quality component testing) have been in use for decades [5]. Although the combination of these two types of evaluation methods can accurately determine the content of the intrinsic quality components of tea leaves, effectively assess the quality of tea leaves, and determine the grade of tea leaves, they still have their shortcomings [6]. The sensory quality assessment method is generally used by a professionally trained assessor to determine the quality of a tea sample by scoring it according to its appearance (tenderness, shape, color, etc.) and intrinsic qualities (liquor color, aroma, taste, infused leaf, etc.) combined with weighting factors. This method is susceptible to subjective factors and the objectivity of the results obtained is not sufficient. The physical and chemical composition method generally requires the use of various sophisticated instruments such as liquid chromatography [7,8], gas chromatographs [9], gas chromatograph–mass spectrometers [10,11], liquid chromatograph–mass spectrometers [12,13], and spectrophotometers [14], which are more expensive and have complex sample pre-treatment processes as well as use toxic and dangerous chemicals. Although the test results are accurate and objective, the method has the drawbacks of cumbersome operation, sample destruction, time-consuming and costly testing, and cannot meet the requirements of the rapid multi-component quantitative testing of product quality and online monitoring of characteristic attributes (grade, origin, etc.) during the distribution and processing of tea.

Obtaining objective, fast, and accurate information on the taste and quality characteristics of tea is the ideal method for the digital control of tea. Currently, the main rapid characterization methods widely used in the field of quantitative analysis and qualitative identification of tea taste quality are near-infrared (NIR) spectroscopy and electronic tongue (ET) sensing techniques. NIR spectra are vibrational spectra of molecular leaps of hydrogen-containing groups (bonds such as O—H, C—H, and S—H) in the non-visible region in the wavelength range 780–2526 nm [15]. The technique is more commonly used in the rapid testing of nutrient content and geographical traceability of tea [16]. In recent years, with the development of small and compact portable NIR devices, which minimize the size and manufacturing costs of equipment, the application scenarios of NIR have been expanded, showing good potential for development. The ET is a category of bionic sensing technology that utilizes a specific array of taste sensors to simulate the human tongue for signal perception and pattern recognition of liquid samples [17]. The technique employs suitable multivariate statistical analysis approaches for data processing of the obtained ET response signals to obtain the taste output results of the multi-sensor array, which objectively and rapidly reflects the taste characteristics of the samples and enables fingerprint identification and classification of the test samples [18]. The method has been studied and applied in wine [19], honey [20], tea [17], and other food industries [21]. Currently, most of the published studies evaluate tea quality with a single portable NIR technique or bionic sensing technology [22,23]. Few studies have fused micro-NIR spectroscopy and ET sensor data to more comprehensively characterize black tea quality and grade.

**Figure 1:**
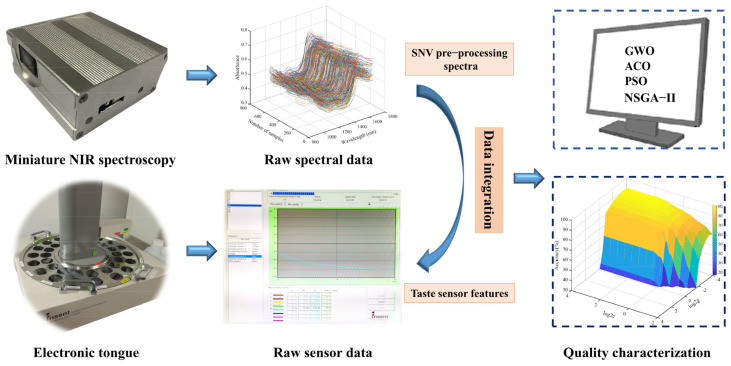
Description of the experimental flow chart.

Thus, the main work of this study is organized as follows: (1) Spectral data and ET response signals from 700 black tea samples of seven grades were collected using a home-made portable NIR spectroscopy system and a commercial ET system, and raw data (i.e., low level) fusion was performed; (2) Four feature selection methods, namely grey wolf optimization (GWO), ant colony optimization (ACO), particle swarm optimization (PSO), and non-dominated sorting genetic algorithm II (NSGA−II), were used to extract features (i.e., mid-level data fusion) on the raw fused data; (3) Support vector machine (SVM), extreme learning machine (ELM) and K-nearest neighbor (KNN) algorithm were employed to construct classification models to determine the quality grade of black tea. The experimental procedure for this study is shown in Figure 1.

## 2. Materials and Methods

### 2.1. Sample Collection

The experimental samples were seven standard grades of Congou Dianhong black tea products, provided by Yunnan Dian Hong Group Co. (Lincang, China). The contents of the main quality components and the organoleptic evaluation results of the samples were obtained through laboratory testing and sensory evaluation panel scoring by the National Standard Method of the People’s Republic of China, respectively, to ensure their quality. The total number of black tea samples was 700, with 100 samples of each grade, in descending order of T, C1, C2, C3, C4, C5, and C6. The grading of black tea samples was mainly determined by their appearance, color, aroma, and flavor when brewed. The tea samples were picked in the spring and autumn of 2019 and 2020. Usually, tea leaves were picked in different seasons to produce a primary processing tea, and the different primary processing teas need to be blended to stabilize the quality of the finished tea product; based on this, grading was carried out. Black tea samples were processed through the processes of withering, twisting, fermentation, and drying of fresh leaves. The samples were dried utilizing a dryer at 90 °C for 30 min. The samples were dried to a moisture content of around 7%. Before data collection, samples were stored in kraft aluminum foil bags and kept in a thermostatic desiccator for three months. Additionally, the single tea sample was tightly knotted, dark brown, and oily, with a uniform appearance. Each tea sample weighed 100 g and was used for multi-sensor signal acquisition and analysis of the data.

### 2.2. Miniature NIR Spectroscopy and Data Pre-Processing

The miniature NIR spectrometer (Figure 1) developed by our group was equipped with a Bluetooth module and could be connected to a Huawei P50 smartphone for spectral data acquisition on the mobile phone. The NIR device had a spectral acquisition range of 900–1700 nm, a resolution of 10 nm, and weighs only 80 g. During the spectral acquisition, each sample was placed into a quartz sample cup for scanning. After ensuring that the bottom of the sample cup was completely covered by the tea sample, the NIRS information of the sample was collected via diffuse reflection mode. A sample spectrum was acquired every 120° of rotation and the average spectral value of the three scans was used as the subsequent spectral data for modeling purposes.

**Figure 2:**
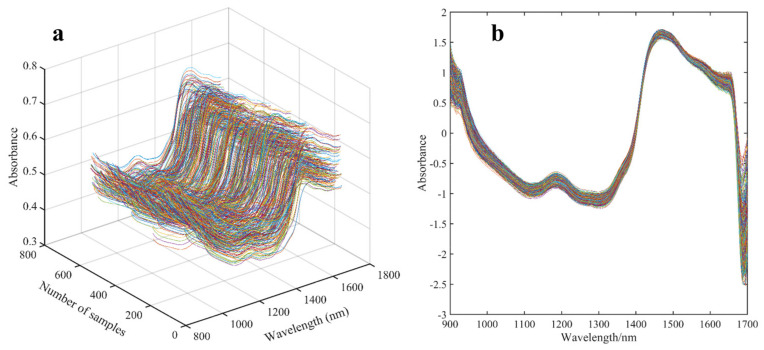
Raw and SNV pre−treatment spectra of samples. (**a**) Raw spectra; (**b**) SNV pre−processing spectra.

As the raw spectra acquired by the NIR instrument are susceptible to the physical properties of the sample, background information, and noise interference, it is necessary to pre-process the raw spectra to obtain high−quality spectral features. The effect of spectral pre-treatment methods on the subsequent modeling performance have been investigated in several published studies by our group [16]. The results of several papers showed that the SNV spectral pre−processing method was an effective way to eliminate the effects of solid particles, scattering, and light range variability on the NIRS spectrum. Therefore, the spectral information from the SNV pre−processing was used as feature data for subsequent data analysis in this study. The original NIR spectra of the samples and the SNV pre−treatment spectra are shown in Figure 2. As can be seen in Figure 2, SNV filters out the noise at the beginning and end of the spectral interval, and the SNV-processed spectral curve is smoother and of better quality.

### 2.3. Electronic Tongue Signal Acquisition

The electronic tongue system, model SA402B, was supplied by Insent Intelligent Sensor Technology of Japan and was equipped with an array of six different taste sensors (i.e., CAO, COO, CTO, AE1, AAE, and GL1). The above sensors could perceive nine taste characteristics: sour, bitter, astringent, fresh, salty, sweet, astringent aftertaste, bitter aftertaste, and richness. Before data collection by the ET sensor system, samples were pre-treated in the following sequence: firstly, 3.00 g of tea samples were placed in standard evaluation cups and bowls and brewed for 5 min with 150 mL of boiling distilled water. The tea extract was then filtered through a triple-layer filter cloth and 35 mL of the tea extract was placed into an ET sample cup and cooled at room temperature for the acquisition of ET response signals. The system data acquisition program was set up as follows: 90 s for cleaning the positive and negative electrodes, 120 s for both washes of the reference solution (30 mM KCl and 0.3 mM tartaric acid), and 30 s for sensor data acquisition. The instrument collected the taste potential signal from the sample solution and output it as the nine taste characteristics described above. In the experiment, each sample was measured four times and the average of the four measurements was used as a reference value for data analysis. The working temperature of the test was maintained at 28 °C in circulating water.

### 2.4. Feature Selection Strategy

To extract feature information from a large number of feature variables related to the exclusive properties of the target substance to be sensed, swarm intelligence optimization algorithms, i.e., PSO, ACO, NSGA−II, and GWO feature variable selection algorithms were introduced to screen ET sensor features and spectra for valid features.

PSO was originally applied as a feature screening method to stimulate social behavior in flocks of birds, fish, and other groups [24]. Considering each bird as each solution of the optimization algorithm in the target space, the population of *N* particles evolved with each iteration and moved towards the optimal solution of the problem according to the principles of the optimization method, with the particles moving through the path optimization of the previous optimal position and the global optimal position to explore the optimal solution for the whole population [25,26]. The evaluation criterion of the algorithm was determined by the optimal cost parameter of the objective function, the value of which was determined in several iterations of the algorithm [27].

ACO was a swarm optimization algorithm based on the naturally evolving foraging behavior of ant colonies [28]. Scientists studying ants foraging for food discovered that they communicated with each other by spreading pheromones [18]. The algorithm was based on the information feedback mechanism of the ant colony to find the shortest path, enabling intelligent search, parameter optimization, and other functions [29].

NSGA−II was a multi-objective genetic algorithm, proposed by Deb et al. [28]. In the structure of the method, in addition to crossover, genetic operators, and mutation, two multi-objective operators (non-dominated sorting and crowding distance) were utilized. The basic idea of NSGA-II was to hierarchically rank populations by the non-dominant sorting of populations, calculate the crowding distances of individuals to maintain population diversity, and obtain an approximate solution when the termination condition was reached [30].

GWO was a population-based optimization algorithm that simulated the leadership hierarchy and hunting strategy of the natural grey wolf [31]. The algorithm was based on the decision making and management of the alpha wolf leading the pack through the process of tracking, rounding up, and attacking the prey, ultimately achieving the global optimal solution for the capture of the prey [32].

### 2.5. Modeling Algorithms

The classification models of linear KNN as well as non-linear ELM and SVM were constructed based on the variable information selected by the above methods and their model performance was compared for merit to explore the best classification model for the sample rank.

The KNN algorithm was based on Euclidean distance to explore similar samples and discriminate between different grades of tea samples, and the performance of this classifier depended heavily on the *K*−value and Euclidean distance chosen [33]. The *K*−value was chosen concerning the minimum prediction error of the best result obtained by the classifier [34]. The method was relatively simple and was considered to be one of the fastest machine learning methods to execute on large datasets with uniformly distributed feature spaces [35].

ELM was a machine learning algorithm for multiple classification and regression based on a single implicit layer feed-forward neural network [36]. The discriminator randomly assigned weighting coefficients connecting the input layer and the implicit nodes. The main optimization of the algorithm was described as follows: (1) determine the number of neurons in the hidden layer and randomly setting the input weights of the nodes in the hidden layer; (2) select the activation function of the neurons in the hidden layer (HL) and calculate the output matrix of the neurons in the HL. Based on the highest recognition rate of the prediction set, the optimal number of neurons in the HL is determined [37].

The SVM method was a common multi-classifier employed in data analysis [38]. The algorithm was based on the principle of structural risk minimization and attempted to improve generalization and reduce expected risk [39]. The SVM discriminator used the radial basis function (RBF) as the kernel function and obtained good predictions by optimizing two parameters (i.e., the penalty parameter *c* and the kernel parameter *g*) [40]. The specific steps of the method were outlined as follows: (1) the leave−one−out method of cross-validation was employed to optimize the core parameters (*c* and *g*); (2) the best parameter pair (*c* and *g*) using the grid search method was determined; (3) the best SVM classification model was built based on the highest output of the correct classification rate (CCR) in the prediction set.

### 2.6. Model Evaluation

In this study, the CCR of the prediction set samples was used as the evaluation criterion for the performance of the model; the higher the CCR value, the higher the prediction accuracy and the better the generalization ability of the model built. All the algorithms for feature selection and qualitative analysis model construction in this work were written by our group and implemented in MATLAB R2020b software (MATLAB Inc., Natick, MA, USA) under Windows 8.1.

## 3. Results and Discussion

### 3.1. Sample Set Division and Principal Component Analysis

**Figure 3:**
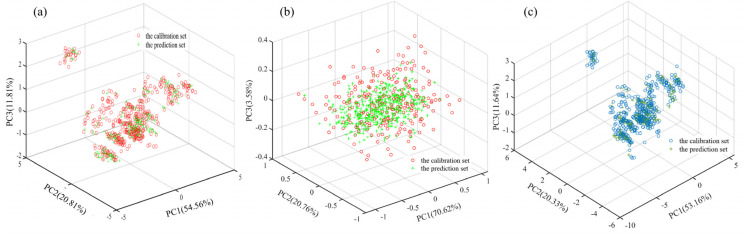
Distributions of Dianhong tea samples from the calibration set and the prediction set in the three−dimensional principal components space. (**a**) ET taste features; (**b**) spectral features; (**c**) data fusion.

The Kennard–Stone sample set partitioning algorithm was introduced to obtain the number of the calibration set and prediction set samples of 467 and 233 in sequence with a partitioning ratio of 2:1. The three−dimensional scatter space distribution of the sample set of Dianhong tea samples based on different feature data is shown in Figure 3. As can be seen from Figure 3, the spatial distribution of the single taste features (ET taste values or NIR spectrum) of the Dianhong samples is more discrete from the sample set of the fused data features, with the distribution of the calibration set samples covering the distribution of the prediction set samples. This result indicated that the distribution of the calibration and prediction set samples was appropriate.

**Figure 4:**
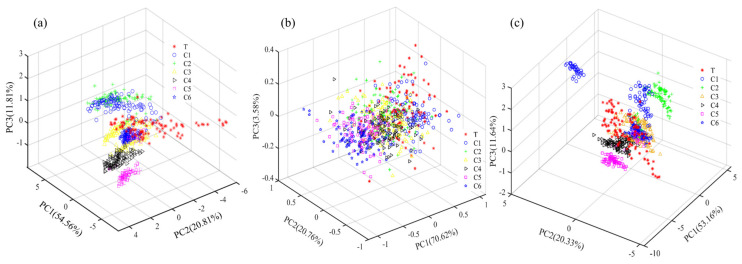
Three−dimensional PC distribution of seven grades of Dianhong tea samples. (**a**) ET taste features; (**b**) spectral features; (**c**) data fusion.

Figure 4 shows the results of the three-dimensional principal component analysis (PCA) distribution of the Dianhong tea samples. The distribution of principal component (PC) scores for single taste characteristics (ET taste values and spectra) and fusion data for the seven classes (T, C1, C2, C3, C4, C5, and C6) of samples showed a high degree of overlap in three-dimensional space between the different quality classes, and it was not simple to distinguish the different classes of samples effectively based on both single and fusion feature data, and there was an urgent need to introduce suitable chemometric methods to achieve effective identification of the quality classes of the samples for testing.

### 3.2. Selection of Taste Characteristic Variables

**Figure 5:**
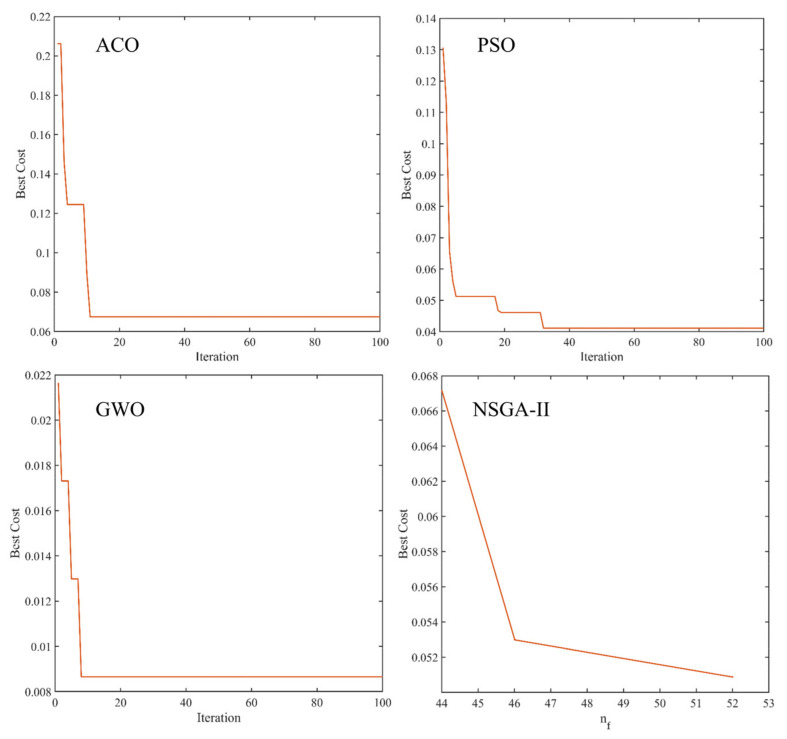
Convergence curves of feature variable selection for different swarm intelligence algorithms.

Four feature selection methods, namely ACO, PSO, GWO, and NSGA−II, were used to feature the low−level fused data from both sensors (ET and NIR spectra). From the convergence curve of feature selection in Figure 5, it can be seen that the above feature variable selection algorithm eventually converged after several iterations to obtain the smallest objective function value as the optimal solution.

The results of the wavelength statistics for the above feature variable selection method over 100 iterations are shown in Table 1. As can be seen from Table 1, the number of sample multi-sensor feature variables extracted by the ACO, PSO, GWO, and NSGA−II algorithms were 12, 15, 83, and 52 in respective order, and the number of variables selected as a proportion of the total number of variables was 5.06% (12/237), 6.33% (15/237), 35.02% (83/237), and 21.94% (52/237). The spectral bands chosen for the above methods were mainly in the long-wave region, and the extracted ET response features all contained astringent aftertaste and umami. In summary, by extracting features from the multi-sensor fusion information of the samples, the computational efficiency and complexity of the subsequent modeling could be further simplified, which was more helpful for the construction of high-quality models.

### 3.3. Results of The Optimal Models

The results of the optimization models built based on ET sensors and spectral features combined with different chemometrics are shown in Table 2. The statistics in Table 2 showed that the order of CCRs for models based on different data using the same modeling approach was roughly low−level fusion data > ET data > NIR data. The CCR of the KNN model based on ET data only was higher than that of the KNN model with fused data. The reason may be that there were more variables in the fused data and the relationships between them were complex, and KNN as a class of linear algorithms was not as advantageous in solving non-linear complex problems. In terms of the performance of models built from the same data source combined with different classification algorithms, the SVM model has the highest CCR. The highest CCR of 92.27% was obtained from the SVM model based on low−level fused data. It could be seen that the modeling performance utilizing fused data outperformed the results of models constructed from single sensor features.

**Figure 6:**
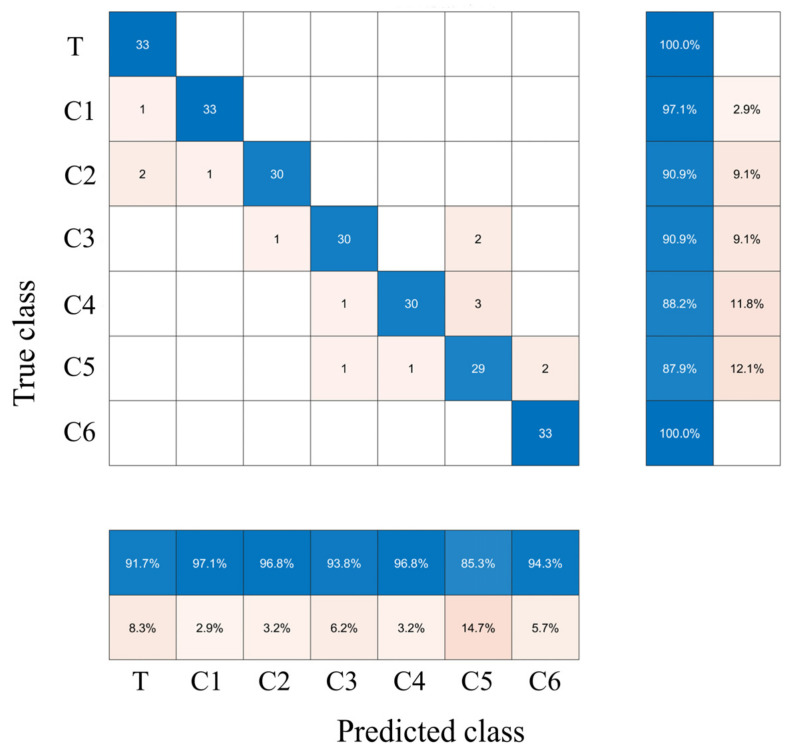
Confusion matrix of the sample distribution of the ACO−SVM model in the prediction process.

The feature fusion data obtained using different variable selection algorithms are the characteristic variables and the prediction results of the models built by combining different machine learning algorithms, as shown in Table 3. The results in Table 3 showed that the accuracy of the prediction set for all models was above 80%. The order of merit of the model performance for the same variable selection method was SVM > KNN > ELM. The CCRs of the ACO−ELM, ACO−KNN, and ACO−SVM models were the highest when analyzed in terms of the model effects of the same modeling approach. It can be seen that the ACO algorithm effectively extracted the feature variables related to the quality grade of Dianhong black tea samples. With fewer variables, the model achieved higher accuracy. The optimal classification model that was built was ACO−SVM with a CCR of 93.56% and only 12 variables were extracted. The ACO−SVM model was more robust than models built from fused data at a low level. To explain in detail the discriminative correctness of the prediction set samples in the ACO−SVM model, the confusion matrix results of the prediction sample distribution are presented in Figure 6. The results of the sample distribution in Figure 6 showed that the CCRs for the seven grades (T, C1, C2, C3, C4, C5, and C6) in the prediction class were 100%, 97.1%, 90.9%, 90.9%, 88.2%, 87.9%, and 100%, respectively. The CCRs for the corresponding seven levels in the true class were 91.7%, 97.1%, 96.8%, 93.8%, 96.8%, 85.3%, and 94.3%, respectively. The plotted confusion matrix gave a visual indication of the classification of the samples. The ACO-SVM discrimination model based on fused features was more effective in evaluating the quality of seven categories of tea samples.

### 3.4. Discussion of the Optimal Models

The characteristic variable features in this study were based on fused data (Miniature NIR data + ET sensor data), and the published papers were all based on a single variable for feature selection [18,41]. The fused data covered a more comprehensive range of information, for which the selection of characteristic variables was beneficial in obtaining valid variables. The results of the selection of feature variables in Table 1 showed that the ACO method obtained the 12 best variables. The number of variables obtained was less than the number of features obtained based on a single spectral information. In published studies based on single spectral data, the performance of the best classification model was also lower than that of the existing ACO−SVM model. Due to the small amount of information from the ET sensor, it was difficult to obtain useful information effectively. Therefore, the stability of the constructed model based on the ET data was insufficient. In terms of the performance of the modeling algorithm, the SVM model had a higher CCR. It had been shown through previous studies that SVM exhibited excellent classification ability in solving non-linear problems [24,42]. In this research, satisfactory results were obtained with the ACO−SVM model.

In the future, the importance of developing small and precise portable NIR equipment to minimize equipment size and manufacturing costs and achieve process control of tea processing quality is an important research direction. In addition, the construction of a cloud-based control system for big data will be an inevitable trend in the development of NIR technology in the field of tea analysis applications.

## 4. Conclusions

NIR spectroscopy and ET sensors can capture the flavor characteristics of tea and thus assess the flavor quality of tea. In this study, a rapid evaluation method for the taste quality (ET, NIR, and fusion data) of Congou black tea was proposed. The effects of different feature wavelength selection methods (ACO, PSO, GWO, and NSGA−II) and intelligent classification algorithms (KNN, ELM, and SVM) on modeling ET features, NIR features, and multi-sensor feature fusion data of Dianhong black tea samples were explored to find the best assessment parameters and recognition models for black tea grade quality. The experimental results showed that the fusion data (ET + NIR) were filtered for features using the feature variable selection algorithms, and the effective fusion recognition models for the quality of Dianhong black tea were established in combination with the classification methods. The discriminative accuracy of the ACO−SVM model based on fused feature vectors was higher at 93.56% compared to the predictive performance of the single sensor data model. It can be seen that the effective fusion of feature data can reflect the intrinsic properties of the samples to be tested more comprehensively, and the fusion based on ET and spectra has good prospects for evaluating the quality of Dianhong black tea.

## Figures and Tables

**Table 1:** Statistical results of different feature variable selection methods within 100 iterations.

Method	Number of Variables	Best Cost	Selected Variables
ACO	12	0.0665	957.03 nm, 977.51 nm, 1004.20 nm, 1024.41 nm, 1286.39 nm,1332.25 nm, 1485.71 nm, 1492.12 nm, 1544.94 nm, 1574.00 nm, astringent aftertaste, umami
PSO	15	0.0411	985.16 nm, 1013.06 nm, 1063.20 nm, 1066.93 nm, 1164.82 nm, 1176.84 nm, 1201.94 nm, 1210.26 nm, 1395.17 nm, 1599.64 nm, 1634.02 nm, bitter aftertaste, astringent aftertaste, umami, saltiness
GWO	83	0.0084	953.17 nm, 964.72 nm, 973.68 nm, 981.33 nm, 985.16 nm, 996.60 nm, 1020.63 nm, 1024.41 nm, 1039.48 nm, 1051.98 nm, 1059.46 nm, 1070.65 nm, 1074.38 nm, 1085.51 nm, 1108.90 nm, 1146.70 nm, 1150.33 nm, 1161.21 nm, 1180.44 nm, 1198.37 nm, 1206.70 nm, 1224.46 nm, 1238.60 nm, 1257.35 nm, 1264.34 nm, 1274.81 nm, 1278.29 nm, 1282.92 nm, 1293.32 nm, 1318.57 nm, 1321.99 nm, 1325.42 nm, 1332.25 nm, 1335.66 nm, 1342.46 nm, 1353.77 nm, 1367.27 nm, 1373.99 nm, 1380.70 nm, 1387.39 nm, 1395.17 nm, 1398.50 nm, 1408.47 nm, 1418.39 nm, 1426.08 nm, 1435.93 nm, 1442.48 nm, 1445.75 nm, 1449.02 nm, 1452.27 nm, 1458.78 nm, 1463.11 nm, 1469.59 nm, 1476.05 nm, 1479.27 nm, 1485.71 nm, 1496.39 nm, 1499.59 nm, 1505.97 nm, 1512.33 nm, 1515.51 nm, 1525.01 nm, 1551.20 nm, 1554.32 nm, 1557.44 nm, 1560.56 nm, 1567.81 nm, 1570.91 nm, 1580.18 nm, 1583.26 nm, 1596.58 nm, 1599.64 nm, 1602.69 nm, 1614.87 nm, 1631.01 nm, 1634.02 nm, 1640.03 nm, sourness, astringency, bitter aftertaste, astringent aftertaste, umami, richness
NSGA−II	52	0.0509	960.88 nm, 969.84 nm, 992.79 nm, 996.60 nm, 1024.41 nm, 1035.71 nm, 1070.65 nm, 1074.38 nm, 1078.09 nm, 1108.90 nm, 1112.58 nm, 1116.25 nm, 1128.47 nm, 1132.12 nm, 1135.77 nm, 1150.33 nm, 1169.64 nm, 1194.79 nm, 1213.82 nm, 1235.07 nm, 1238.60 nm, 1246.82 nm, 1250.33 nm, 1325.42 nm, 1339.06 nm, 1353.77 nm, 1363.90 nm, 1391.84 nm, 1418.39 nm, 1429.37 nm, 1432.65 nm, 1435.93 nm, 1442.48 nm, 1445.75 nm, 1449.02 nm, 1472.82 nm, 1488.92 nm, 1541.81 nm, 1554.32 nm, 1570.91 nm, 1589.42 nm, 1596.58 nm, 1605.74 nm, 1611.83 nm, 1617.91 nm, 1620.94 nm, 1623.97 nm, sourness, bitter aftertaste, astringent aftertaste, umami, sweetness

**Table 2:** Results of the optimal models based on ET sensors and spectral features combined with different chemometrics.

Data	Model	Parameters	CCR/%			
			Calibration Set		Prediction Set	
ET	ELM	nn ^a^ = 45	358/467	76.66	178/233	76.39
	KNN	PCs = 4, *K* = 1	408/467	87.37	201/233	86.27
	SVM	*c* = 16, *g* = 5.66	422/467	90.36	210/233	90.13
NIR	ELM	nn ^a^ = 38	328/467	70.24	150/233	64.38
	KNN	PCs = 3 *K* = 3	333/467	71.31	152/233	65.24
	SVM	*c* = 16, *g* = 0.062	425/467	91.01	198/233	84.98
Low−level Fused data	ELM	nn ^a^ = 97	376/467	80.51	185/233	79.40
	KNN	PCs = 6, *K* = 5	370/467	79.23	182/233	78.11
	SVM	*c* = 16, *g* = 0.062	455/467	97.43	215/233	92.27

^a^ Number of hidden layer neurons.

**Table 3:** Results of optimization models based on fused data and different chemometric methods.

Model	Parameters	CCR/%			
		Calibration Set		Prediction Set	
GWO−ELM	nn ^a^ = 73	397/467	85.01	196/233	84.12
GWO−KNN	PCs = 5, *K* = 3	408/467	87.37	203/233	87.12
GWO−SVM	*c* = 16, *g* = 0.25	450/467	96.36	216/233	92.70
ACO−ELM	nn ^a^ = 83	398/467	85.22	197/233	84.55
ACO−KNN	PCs = 9, *K* = 7	421/467	90.15	210/233	90.13
ACO−SVM	*c* = 16, *g* = 0.70	448/467	95.93	218/233	93.56
PSO−ELM	nn ^a^ = 97	386/467	82.66	189/233	81.12
PSO−KNN	PCs = 8, *K* = 7	421/467	90.15	208/233	89.27
PSO−SVM	*c* = 11.31, *g* = 2.83	440/467	94.22	217/233	93.13
NSGA−II−ELM	nn ^a^ = 73	394/467	84.37	195/233	83.69
NSGA−II−KNN	PCs = 6, *K* = 5	412/467	88.22	205/233	87.98
NSGA−II−SVM	*c* = 16, *g* = 0.35	455/467	97.43	216/233	92.70

^a^ Number of hidden layer neurons.

## Data Availability

Not applicable.
